# The Role of Immunotherapy in Multiple Myeloma

**DOI:** 10.3390/ph9010003

**Published:** 2016-01-14

**Authors:** Mehmet Kocoglu, Ashraf Badros

**Affiliations:** Marlene and Stewart Greenebaum Cancer Center, University of Maryland Medical Center, Baltimore, MD 21201, USA; abadros@umm.edu

**Keywords:** myeloma, immunotherapy, vaccines, tumor antigens, antibodies, CAR-T, adoptive cell therapy

## Abstract

Multiple myeloma is the second most common hematologic malignancy. The treatment of this disease has changed considerably over the last two decades with the introduction to the clinical practice of novel agents such as proteasome inhibitors and immunomodulatory drugs. Basic research efforts towards better understanding of normal and missing immune surveillence in myeloma have led to development of new strategies and therapies that require the engagement of the immune system. Many of these treatments are under clinical development and have already started providing encouraging results. We, for the second time in the last two decades, are about to witness another shift of the paradigm in the management of this ailment. This review will summarize the major approaches in myeloma immunotherapies.

## 1. Introduction

Multiple myeloma (MM) is a malignant disease of plasma cells that accounts for about 1% of new cancers and 10% of all hematological malignancies. It affects elderly patients with a median age of 67 years at presentation. The introduction of several novel agents and targeted therapies has significantly improved outcomes for patients with MM. Although few remain in long-term remissions, the majority of patients relapse and die from the disease, usually with a resistant clone. Immune therapy, in the context of an allogeneic graft, has for years been considered the only curative approach for the disease. Unfortunately, allografts are associated with high mortality and overall poor quality of life due to the development of graft *versus* host disease. Several attempts to control the disease using variety of immune modulation have been made. In the following review, we will summarize the current knowledge of the immune dysfunction in MM and the development of various immune modalities to eradicate the disease. We will review the current uses of immunomodulatory drugs, monoclonal antibodies, various vaccination strategies, autologous activated NK and T cells, engineered T cells and the evolving role of checkpoint inhibitors.

## 2. Immune Dysregulation in Multiple Myeloma

It is well established today that all MM patients have a pre-existing none-malignant stage known as monoclonal gammopathy of unknown significance (MGUS) [1]. The mechanism of progression is not solely limited to genetic mutations in the plasma cells but to alterations in the marrow microenvironment and more importantly to loss of immune surveillance.

Although myeloma is primarily a disorder of the B cell lineage, the T cell compartment is frequently affected [2]. This defect is characterized by a significant reduction in the absolute number of CD4 cells whereas the numbers of CD8 lymphocytes remain normal, leading to a decreased CD4/CD8 ratio [2]. In fact loss of tumor specific T cells of CD4, CD8 and NK T cell subsets is a hallmark for progression from MGUS to MM [3]. The balance between regulatory T cells (Treg) and T helper (Th) 17 cells is essential for maintaining anti-tumor immunity in MM [4]. Tregs play an important role in the preservation of self-tolerance and modulation of overall immune responses against infections and tumor cells. In MM patients, Tregs seem to contribute to myeloma-related immune dysfunction. Th17 cells protect against fungal and parasitic infections and participate in inflammatory reactions and autoimmunity. The interplay of TGF-β and IL-6, expressed at high levels in the bone marrow of myeloma patients, may affect generation of Th17 cells both directly or via engagement of other pro-inflammatory cytokines and thereby modulate antitumor immune responses. The balance between Tregs and Th17 cells seems to be skewed towards Th17 cells [5]. This has been affected by IL-6, tipping the balance between reciprocal developmental pathways of Tregs and Th17s towards Th17 route [6]. The result is significant immune deficiency in MM.

MM immune dysregulation affects other aspects of the immune system as well, directly affecting antigen presentation and up-regulation of inhibitory antigens that promotes immune escape and growth advantage for malignant clones. On the antigen presenting side, elaborate studies on different aspects of dendritic cell (DC) biology have revealed somewhat conflicting results. Some studies have reported defects in peripheral blood DCs such as decreased numbers of circulating peripheral blood monocytes, plasmacytoid DCs (pDCs) and myeloid DCs (mDCs), lower expression levels of both MHC class II (HLA-DR) and costimulatory molecules (CD40, CD80) as well as decreased alloreactivity against lymphocytes particularly in the setting of IL-6 inhibition [7]. Other studies showed phenotypically and functionally quasi-normal DC biology from peripheral blood and marrow of MM patients and suggested a contributory role of tumor microenvironment to the previously described defects. This was suggested by elevated IL-6 and VEGF levels in the bone marrow sera in MM patients which lead to an inhibition of induction and maturation of DCs [8]. It is also intriguing to detect MM specific antibodies against tumor antigens (e.g., SOX2) at higher concentrations in MGUS states compared to MM [5]. The direct effects of alterations of immune system may clinically be observed by increased risk of infections in myeloma patients. Kristinsson *et al.* have demonstrated via a population based study that the infection risk even at preclinical stage ie MGUS was increased two folds in 5 and 10 year follow up periods including both bacterial and viral infections [9].

## 3. Immunotherapy in Multiple Myeloma

Standard treatments for MM include standard and high-dose chemotherapy, proteasome inhibitors and IMiDS which usually are given in combinations in conjunction with corticosteroids in the absence or presence of stem cell support. These treatments have radically changed the disease history and improved overall response rates and survival. However, the disease remains incurable and relapse is inevitable in majority of patients. Immunotherapy for 30 years, in the form of an allogeneic stem cell transplant (all-SCT), has been the only treatment modality associated with long-term complete remissions and possibly cures in MM [10]. An effect attributed to the “graft-*versus*-myeloma”; a proof of principle that the immune system can eradicate a malignant clone has unfortunately limited clinical success primarily due to lack of a predictable uniform and potent response as well as to complications of the procedure including high treatment related mortality [11,12].

Several innovative approaches to enhance the immune system to fight MM and stimulate a “host-*versus*-myeloma” effect that can benefit the majority of MM patients have been explored. In the following paragraphs we will review immunotherapies as a promising strategy in eliminating the malignant clone in MM focusing on three approaches:

(1) Reverse tumor mediated immune paralysis
  - Immunomodulatory drugs (e.g., IMiDs).
  - By blocking inhibitory molecules or, alternatively, activating stimulatory molecules, these treatments are intended to enhance pre-existing anti-myeloma immune responses. (e.g., immune checkpoint inhibitors).
  - Cytokines (e.g., interferon and GM-CSF, Siltuximab, an anti-IL-6 antibody and ALT-803, an interleukin 15 agonist).
(2) Stimulate myeloma specific immune responses
  - MM Vaccines (e.g., dendritic cell based, peptide based antibodies).
  - Adoptive T Cell Transfer (focused on genetically modified T cells to target various MM antigens. (e.g., chimeric antigen receptor (CAR) T cells using BCMA and CD-19 or TCR cells engineered to target the NY-ESO-1 antigen).
(3) Selectively eliminate the malignant clone.
  - Monoclonal antibodies (e.g., daratumumab, an antibody against CD38 and elotuzumab, an antibody targeting SLAMF7).

### 3.1. Immunomodulatory Drugs (IMiDs)

IMiDs is a class of drugs that directly affect MM cells and bone marrow microenvironment leading to modulation of cytokines, inhibition of angiogenesis, and augmentation of immune effector numbers and function (T-cell, NK-cell, and NK-T). Recently interaction of IMiDs with cereblon, a ubiquitin ligase component responsible for substrate binding was shown to be crucial for direct cytotoxic and immune related effects [13]. T-cell co-stimulation by lenalidomide or pomalidomide is cereblon dependent and employs two downstream transcription factors, Ikaros (IKZF1) and its paralog Aiolos (IKZF3) [13]. Lenalidomide and pomalidomide also inhibit Treg proliferation. In addition to their effects on T cells, IMIDs are shown to augment NK cell antibody-dependent cellular cytotoxicity (ADCC) via increasing NK cell FasL and Granzyme B expressions [14]. These properties make IMiDs perfect companions to the clinical activities of monoclonal antibodies (mAbs) and to the immune based cellular therapies [15]. The synergistic effects of these combinations are discussed in more detail below in the context of each treatment.

### 3.2. Vaccination Strategies

Two separate vaccination approaches have been developed. The first approach is peptide-based vaccines. The pioneer of these approaches, use of idiotype proteins (Id), an attractive concept as it is, did not meet expectations as potential targets possibly secondary to poor immunogenic nature of the protein as well as low expression of these proteins on the plasma cell surface [16]. There have been efforts to increase immunogenicity via the use of keyhole limpet hemocyanin (KLH), granulocyte macrophage colony stimulating factor (GM-CSF), tetanus toxoid fragments and DCs. In the case of idiotypic proteins, a study investigating the benefit of using idiotype pulsed DCs generated from CD34 progenitors showed good tolerability and safety in 11 patients, yet poor biologic response were seen in about half, some with increase in humoral response and even less with T cell activity [17]. Again, a phase II study investigated stimulation of dendritic cells *ex vivo* with idiotypic proteins (Mylovenge) in a way similar to the FDA approved prostate cancer treatment sipuleucel (Provenge) reported an almost 2 year overall survival benefit yet had study limitations [18]. There are ongoing active Id peptide vaccination trials in various forms such as idiotype pulsed DCs and Id-KLH. (Table 1).

On the other hand, subsequent identification of tumor associated antigens such as MAGE, NY-ESO1, WT-1, RHAMM-R3 and XBP-1 and their use as targets was able to generate cellular responses when used individually and/or in combination in preclinical studies. Clinically, initial results using a single peptide based vaccine demonstrated that such vaccines can be used with few adverse effects and can elicit immune responses but with modest effect on disease control [19]. Particularly there is a focus on cocktail of fragments of peptides that have abundant expression on myeloma cells. In a preclinical study that uses T cells from myeloma patients, peptide specific cytotoxic T lymphocytes were successfully generated using XBP-1, CD138 and CS1 as immunogens [20]. This was more recently replicated using cells from smoldering myeloma patients which also showed an enrichment in the effector memory CD8 T cell subset suggesting potentially durable responses [21]. Currently active peptide vaccination trials in myeloma include WT1, hTERT, MAGE-A3 with NY-ESO-1, MAGE-A3 with AS15 and MUC1 (Table 1). Efforts to enhance the immunogenicity of these vaccines, attempted combination with T cell therapy (see below), although there were impressive immune responses the clinical outcome was lacking.

**Table 1:** Selected tumor associated antigen targets under clinical trials.

Setting	TAA Target	Platform/Adjunct Treatment	Phase	Status	Identifier
MM (ISS I,II,III)	WT1	ASCT	Not provided	R	NCT01827137
Advanced myeloma	hTERT	ASCT	I/II	U	NCT00834665
High risk myeloma	MAGE-A3 and NY-ESO-1	DTPACE with ASCT	II/III	C	NCT00090493
Various malignancies	NY-ESO-1	Resiquimod and/or Poly-ICLC	I/II	C	NCT00948961
Symptomatic ISS stage I,II,III myeloma	MAGE-A3 and AS15	After ASCT	I	ONR	NCT01380145
Early stage (ISS—I) myeloma	Id (DC based )	n/a	I	C	NCT00988312
Late stage (ISS—II,III) myeloma	Id pulsed DC	Tandem auto/allo SCT	I/II	C	NCT00186316
MM >12 months of therapy	Id-KLH	CD3/CD28 activated T cells ASCT	II	R	NCT01426828
MM	MUC1	GM-CSF	II	W	NCT00162500
Various malignancies	MUC1	hGM-CSF	I/II	C	NCT01232712

TAA: Tumor associated antigen; ASCT: Autologous stem cell transplantation; R: Recruiting; U: Unknown; C: Completed; ONR: Ongoing, not recruiting; W: Withdrawn prior to enrollment; n/a : not applicable.

The second vaccination approach involves dendritic cell/myeloma cell fusion (DC/MM). This strategy takes advantage of the ability of the DC to present several antigens from the cell to the host [22]. DC/MM fusions were evaluated in a phase I/II clinical trials [23,24]. In both studies vaccination with DC/MM fusions were well tolerated and stimulated tumor specific immunity as evidenced by expansion of tumor reactive CD4 and CD8 T cells and induction of tumor specific antibody responses. In the second trial, infusion of DC/MM cells on day 100 after transplant was associated with depletion of regulatory T cells. Interestingly, a quarter of patients with partial response (PR) converted to complete response (CR) after vaccination suggesting that vaccine induced immune responses eliminated minimal residual disease (MRD) [24]. This phase II study is the bases of the ongoing (CTN 1401) trial.

### 3.3. Antibody Therapies

The development of effective cytotoxic mAb therapies in MM has been hindered by the lack of distinctively and constitutively expressed target molecules on malignant plasma cells. Studies early after the turn of millennium demonstrated only minimal activity of anti-CD20 rituximab which is expressed on 20% of plasma cells. This was followed by several mAbs (against CD40, IGF-1R, CD56, CS1, CD138, CD74, IL-6R, CD38, TRAIL-R1). For the purpose of this review we will focus on 2 mAbs that have already demonstrated promising clinical activity in MM.

#### 3.3.1. Elotuzumab; Anti-CS1 (SLAMF7) Antibody

CS-1 is a transmembrane glycoprotein expressed on normal and malignant plasma cell membranes as well as on NK cells [25]. An IgG1 antibody targeting CS1, elotuzumab, has shown impressive *in vitro* activity against myeloma cells, killing myeloma cells via antibody dependent cellular cytotoxicity (ADCC) while using the same receptor for activation of NK cells [25,26]. This antibody does not have complement dependent cytotoxicity (CDC) [27]. The initial single agent phase I trial showed no clinical activity in a heavily pre-treated population [28], however, in conjunction with revlimid and dexamethasone, elotuzumab had an impressive 82% objective clinical response in relapsed patients after a median of three lines of prior treatment; median time to progression was still not reached after 16.4 months of follow-up [29]. Recently, a large phase III study (ELOQUENT-2) involving 600 relapsed MM patients confirmed the efficacy of the combination of elotuzumab plus lenalidomide and dexamethasone compared to lenalidomide and dexamethasone alone; progression free survival was 68% and 41% at 1 and 2 years (compared to 57% and 27% in controls) [30]. Interestingly, elotuzumab also showed activity against disease with high risk cytogenetic features such as t(4;14) and del(17p) as well as, to a lesser extent, +1q21 [30]. It is also important to note that even though prior treatment with lenalidomide was allowed in this study, only 10% of the enrolled population had previously been treated with lenalidomide. Combination trials with proteasome inhibitors showed promising results as well, albeit to a lesser degree. Several phase I studies are underway exploring roles of elotuzumab along with anti-KIR antibodies (lirilumab—BMS-986015) or anti-CD137 (urelumab—BMS 663513).

#### 3.3.2. Daratumumab; Anti-CD38 Antibody

**Figure 1:**
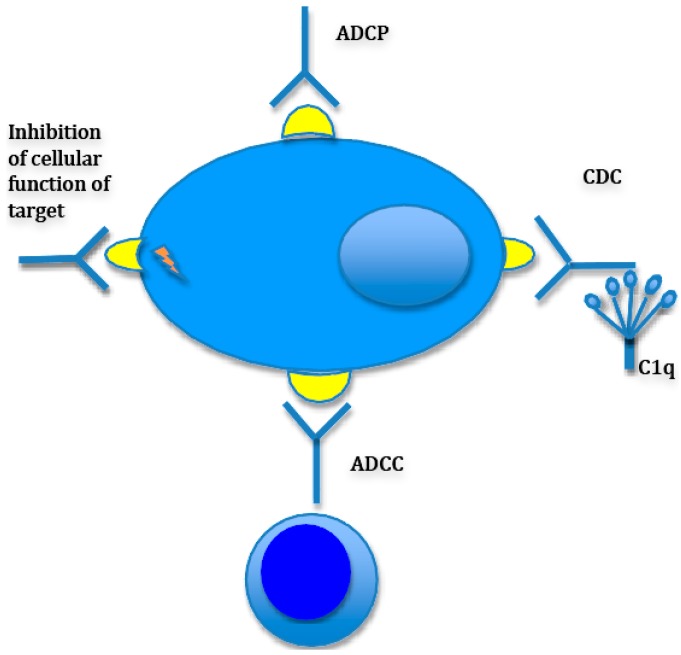
Mechanisms of antibody-mediated myeloma cell killing. Antibodies kill myeloma cells via different mechanisms, some more contributory to the killing effect than the others. There is also variability between antibodies with regards to mechanisms. For instance, while daratumumab was shown to possess all the above mechanisms of action while anti-CS1 antibody elotuzumab is devoid of CDC function. (ADCC: Antibody dependent cellular cytotoxicity; CDC: Complement dependent cytotoxicity; ADCP: Antibody dependent cellular phagocytotoxicity).

CD38 is a type II transmembrane glycoprotein with multiple proposed functions in cell adhesion, signaling and enzymatic (cellular nucleic acid metabolism) activity and is expressed on a multiple hematopoietic and non-hematopoietic cell types. Among many hematopoietic cells that harbor this antigen are medullary thymocytes, subpopulations of both activated B and T lymphocytes, NK cells and dendritic cells [31]. Daratumumab is a fully human IgG1κ monoclonal antibody directed against CD38, that has shown activity against myeloma cells in preclinical models. Among the proposed mechanisms of action of daratumumab, in addition to well described CDC and ADCC are antibody dependent phagocytosis (ADCP), induction of autophagy/apoptosis as well as loss of enzymatic activity (Figure 1).

In phase I/II study recently published by Lokhorst *et al.*, impressive clinical responses were seen in heavily pretreated patient population with 64% double refractory to PIs and IMiDs and had undergone ASCT in 76% [32]. Daratumumab as a single agent yielded 36% overall response rate in 16 mg/kg arm and remarkably, in the responder group, 65% remained progression free in 12 months.

Two additional antibodies that target CD38 are SAR650984 (isatuximab) and MOR03087 (MOR202; MOR) which are under clinical development as monotherapy or in various combinations.

Several new mAbs are under development for various cell member targets and others are in early stages but few are worth mentioning. The first is B cell maturation antigen (BCMA), a protein of TNF receptor superfamily which is crucial for long term survival of plasma cells through its binding B cell activating (BAFF) and proliferating (APRIL) factors [33]. Several studies assessing mAb against BCMA and antibody drug conjugates are underway. Antibodies against CD138 (syndecan) seems limited by the soluble forms of CD138, however when an mAb is combined with tubulin polymerization inhibitor maytansinoids, there were significant preclinical as well as early clinical activities in phase I trial [34]. Antibodies targeting CD56 and CD74 are in early stages of clinical development.

#### 3.3.3. Bispecific T Cell Engagers (BiTEs)

A new area of antibody research has recently focused on bispecific T cell engagers (BiTEs) that combine specificities of two antibodies by simultaneous binding to multiple epitopes, one of which involves the engagement and activation of T cells via their CD3 molecules [35] The first bispecific antibody generated specifically against myeloma was developed by combining single-chain variable fragments (ScFv) of a mAb that binds normal and malignant plasma cells (Wue-1) and a mAb against CD3, forming BiTE product (bscWue-1 × CD3) [36,37,38]. This led to design and development of other BiTEs. A promising bispecific engager that is currently under clinical investigation targets BCMA via a defucosylated antibody (with the goal of increased binding to Fc receptors) that is conjugated to the monomethyl aurastatin F (MMAF, GSK2857916). This antibody is currently being investigated in a Phase I open-label dose escalation study in relapsed/refractory setting of which clinical outcomes are eagerly awaited. (NCT02064387) [39].

### 3.4. Immune Checkpoint Inhibitors (PD-1/PD-L1 Axis)

Immune checkpoint inhibitors targeting PD-1 (pidilizumab, pembrolizumab and nivolumab) on T cells or its cognate ligand, PD-L1 on tumor cells have established activities in many different types of cancers [40]. Whereas the initial postulated mechanism of action of the checkpoint blockade was primarily via the engagement of T cells that are being regulated by peripheral tolerance, a growing body of evidence suggests important roles of antigen presenting cells and activation of NK cells [41]. The preliminary results of a Phase I study with nivolumab that enrolled multiple hematologic malignancies reported disappointing results with no objective responses according to IMWG criteria in 27 myeloma patients, however 67% of patients remained in stable disease in a population of which two thirds of MM patients were heavily pretreated with more than 3 lines of treatment [42]. Another PD-1 antibody, pidilizumab (CT-011) was investigated via a phase I trial that enrolled 17 patients with various hematological malignancies and has yielded stable disease in the single myeloma patient in the cohort, however, with a durable response for > 13 months [43]. There are also clinical studies looking into safety and efficacy of pembrolizumab, primarily in combination with IMiDs. Recently, preliminary results of a phase II trial with pembrolizumab with pomalidomide was presented and reported 50% objective response, including near complete and very good partial responses in a double refractory RRMM patient population [44]. Currently there are several clinical studies investigating the use of immune checkpoint inhibitors in various combinations in MM, majority being PD-1 trials. (Table 2) In addition, there is a growing interest in its cognate molecule, PD-L1 as this is the part of the signaling pathway that is harbored on the tumor itself and at least in theory has the additional potential for ADCC. This ligand was shown to be expressed on malignant plasma cells [45]. In addition, PD-L is also expressed on other cells as well such as on plasmacytoid DCs (pDC) and myeloid derived suppressor cells (MDSC) both of which play role in immunosuppressive state in myeloma. The use of PD-L1 antagonists is also being explored in the context of IMiD presence or absence (Table 2).

**Table 2:** Selected immune checkpoint blockers under clinical trials.

Setting	PD1 Antibody	IMiD	Additional Intervention	Phase	Status	Identifier
NDMM	Pembrolizumab	Lenalidomide	n/a	III	R	NCT02579863
RRMM	Pembrolizumab	Lenalidomide	n/a	I	R	NCT02036502
RRMM	Pembrolizumab	Pomalidomide	n/a	I/II	R	NCT02289222
RRMM	Pembrolizumab	Pomalidomide	n/a	III	R	NCT02576977
RRMM	Pidilizumab	Lenalidomide	n/a	I/II	R	NCT02077959
Post ASCT	Pembrolizumab	Lenalidomide	n/a	II	R	NCT02331368
RRMM	Nivolumab	n/a	Ipilimumab Lirilumab	I	R	NCT01592370
Post ASCT	Pidilizumab	n/a	DC/MM	II	ONR	NCT01067287
Locally advanced/metastatic solid tumors or hematological malignancies	MPDL3280A	n/a	n/a	I	R	NCT01375842
MM	MPDL3280A	Lenalidomide	n/a	Ib	R	NCT02431208

NDMM: Newly diagnosed multiple myeloma; RRMM: Relapsed refractory multiple myeloma; ASCT: Autologous stem cell transplantation; R: Recruiting; U: Unknown; C: Completed; ONR: Ongoing, not recruiting; W: Withdrawn prior to enrollment; n/a : not applicable.

### 3.5. Adoptive T Cell Therapies (ACT)

Early studies suggested that early lymphocyte count recovery after auto-SCT correlated with improved disease control [46,47]. Several trials involved *ex vivo* co-stimulation of autologous T-cells via immunomagnetic beads (anti-CD3/CD28 beads) which in the presence of interleukin-2 led to significant activation and expansion of T cells. Infusion of these cells after myeloablative bone marrow conditioning and autologous stem cell transplantation led to early lymphocytosis. In trials using peripheral blood there is no clear evidence for a tumor specific T cell enhancement effect and no impact on outcome. This was probably related to nonspecific stimulation of the entire T cell repertoire including regulatory T cells. However, it was also noted during early trials that vaccination responses were enhanced in patients receiving *ex vivo* expanded T cells. This led to a series of trials using various antibodies (Idiotype, MAGE, hTERT, Survivin) all of which were associated with enhanced antibody and cellular immunity against the vaccine. In the MAGE antibody trial, vaccine-specific cytokine-producing T cells were detected in 19 of 25 patients (76%), however, the clinical outcomes were not correlated with the generated immunity [48]. This may be a reflection of the tumor heterogeneity and/or immune escape mechanisms.

Recently, Borrello and colleagues have shown for the first time that expanding the subset of marrow-infiltrating T lymphocytes (MILs) can led to clinical antitumor immunity [49]. The results are encouraging but experience with ACTs awaits confirmation in a larger trials.

### 3.6. Chimeric Antigen Receptor (CAR) T Cells

Single chimeric antigen receptor T-cell based therapy (CART), represents a huge leap in immune therapy. CART cells, constructed by fusing the single chain variable fragment (scFv) of a monoclonal antibody (mAb) specific for a surface antigen with an intracellular signaling domain have shown activity in several CD-19 related disease. The MHC-independent tumor recognition, *in vivo* expansion and memory cell generation confers these cells a clear advantage over naked antibodies or adoptively transferred tumor-reactive T cells. A successful example of CD19 targeted CAR—T cell approach was recently published suggesting activity of this therapy [50]. Even though plasma cells do not have a strong CD-19 expression, Garfall *et al.* have observed a low, nevertheless a more frequent expression than previously reported on malignant plasma cells and targeting this population via the use of CTL019 cells (lentivirus transduced autologous T cells harboring CD3-zeta/CD137 based anti-CD19 chimeric receptor), reported remission in a 43 year old with 9 prior lines of treatment [50]. In this report, this therapy was well tolerated without cytokine release syndrome. They report on treating another 9 patients with more than half being in remission [50]. Again, another example of this strategy that employs an ontogenetically later target ie BCMA-directed lentiviral transduced CAR clinical trials, with restricted and consistent expression pattern of the target antigen are encouraging [51]. Additional attempts using other targets in MM have been less successful and have been attributed to the lack of expression of the target antigen on relevant clones and there are yet others not attempted due to low expression. However, as in the case of CD19 directed CARs, there may be a role of this strategy even for weakly expressed antigens or perhaps due to dynamic nature of surface antigen expressions. At the time being, it remains unclear whether simultaneous targeting of multiple antigens (such as CD38, CS1, BCMA, CD138, *etc.*) will be needed to eliminate the malignant clone.

### 3.7. TCR Transgenic T Cells

Infusion of auto-engineered T cells with affinity enhanced TCR specific for a common peptide shared between two cancer testis antigens (NY-ESO-1 and LAGE-1) in MM was recently reported [52]. Patients needed to have HLA-A2 and their MM cells expressed NY-ESO-1 and/or LAGE-1. A total of 24 patients were treated and at last follow up, eight remained in remission with a median PFS of 19.1 months and median OS of 32 months. The duration of response is reasonable and seems better than would be expected in this population. Initial laboratory data suggest that the infused cells remain functional in the absence of IL-2 and without exhaustion for up to one year. Relapse patients were both antigen negative (indicating mutational change in the target) and positive (probably reflecting T cell exhaustion). However, as these cells are HLA dependent, this approach has a limited utility compared to CART cells.

### 3.8. Cytokines

#### 3.8.1. Interleukin–6 (IL-6)

Interleukin-6 (IL-6) is a cytokine that has long been under spotlight in myeloma since late 80s as a driver for myeloma which raised the question of benefit of targeting this cytokine for theurapeutic purposes [53]. A phase I/II dose escalation study with chimeric monoclonal anti-IL-6 antibody (CLB IL6/8) resulted in a reduction of endogeneous production, an effect that was attributed to the blockage of a positive feedback loop [54]. A phase II randomized, double blind, placebo controlled study where another anti-IL-6 antibody, siltuximab (CNTO 328), in combination with bortezomib, was compared to bortezomib and placebo combination. In this study, addition of siltuximab to bortezomib in RRMM failed to improve PFS or OS [55]. There are multiple other studies looking at different combinations of IL-6 blockade in conjunction with other standards of care such as bortezomib, VD, VRD and VMP (Table 3).

#### 3.8.2. Interleukin-15 (IL-15)

Interleukin-15 (IL-15) is a cytokine that has a critical role in CD8 memory cell and NK cell development, proliferation and activation, making it an attractive target. A complex with superagonistic activity against this cytokine, ALT-803 was recently developed which in preclinical models was able to employ specifically CD8 memory cells [56]. Whereas NK cells are also activated, the anti-myeloma activity was independent of this activation. A phase I/II study looking into the safety and efficacy of ALT–803 in RRMM is currently under investigation whereas another one is exploring its role in the setting of post-allogeneic SCT relapse in hematological malignancies (Table 3).

**Table 3:** Selected cytokine blocking drugs under clinical trials.

Setting	Cytokine	Inhibitor	Additional Intervention	Phase	Status	Identifier
NDMM	IL-6	CNTO 328	VMP combination	II	C	NCT00911859
MGUS, SMM, indolent MM	IL-6	CNTO 328	Cardiac functions	I	C	NCT01219010
High risk smoldering	IL-6	CNTO 328	n/a	II	ONR	NCT01484275
RRMM	IL-6	CNTO 328	VD	III	W	NCT01266811
NDMM	IL-6	CNTO 328	VRD	Ib/II	C	NCT01531998
MM, NHL, Castleman’s disease	IL-6	CNTO 328	n/a	I	C	NCT00412321
RRMM	IL-6	CNTO 328	Dexamethasone	II	C	NCT00402181
RRMM	IL-6	CNTO 328	Bortezomib	II	ONR	NCT00401843
RRMM	IL-15	ALT-803	n/a	I/II	R	NCT02099539
Post allogeneic stem cell transplantation	IL-15	ALT-803	n/a	I/II	R	NCT01885897

NDMM: Newly diagnosed multiple myeloma; RRMM: Relapsed refractory multiple myeloma; NHL: Non-Hodgkin lymphoma; VMP: Bortezomib, Melphalan, Prednisone; VR: Bortezomib, Lenalidomide, Dexamethasone; R: Recruiting; U: Unknown; C: Completed; ONR: Ongoing, not recruiting; W: Withdrawn prior to enrollment; n/a: not applicable.

## 4. Future

Myeloma is a disease which has seen four decades of treatment with alkylating agents and steroids with barely any improvement in response statistics. Following the advances in basic and translational research, introduction of novel agents, particularly combination therapies, improved indicators of quality of life and survival significantly. With a median survival of over 5 years and counting, finally our attentions are turned to different strategies to obtain deeper and more durable remissions by starting treatment at asymptomatic stages and incorporating minimal residual disease (MRD) to IMWG response criteria. However, it is important to realize that even at the MRD stage, one still has about one in 10^−5^ malignant plasma cell burden which we are not currently able to treat [57]. It is possible that the aforementioned immune therapies may be the long sought pieces of puzzle. Paved way by the anti-CD20 treatment rituximab, many different monoclonal antibodies have been designed, developed and have already started shifting the paradigm in the treatment of cancers. This is particularly true for myeloma where various immune therapies are being investigated with exciting results. mAbs, vaccines, IMiDs, checkpoint inhibitors, CARTs and TCRs bear great theurapeutic potential to overcome effects of immune suppressive cytokines and accessory cells in the BM microenvironment, restore host CD4, CD8, and NK cell anti-tumor immunity, and improve patient outcome in MM and with their acceptable toxicity profiles are prime candidates for not only the treatment on relapsed refractory patients but also in newly diagnosed and even in preclinical disease.
